# Artemisinin resistance containment project in Thailand. (I): Implementation of electronic-based malaria information system for early case detection and individual case management in provinces along the Thai-Cambodian border

**DOI:** 10.1186/1475-2875-11-247

**Published:** 2012-07-29

**Authors:** Amnat Khamsiriwatchara, Prayuth Sudathip, Surasak Sawang, Saowanit Vijakadge, Thanapon Potithavoranan, Aumnuyphan Sangvichean, Wichai Satimai, Charles Delacollette, Pratap Singhasivanon, Saranath Lawpoolsri, Jaranit Kaewkungwal

**Affiliations:** 1 Center of Excellence for Biomedical and Public Health Informatics (BIOPHICS), Faculty of Tropical Medicine, Mahidol University, Bangkok, Thailand; 2 Bureau of Vector-borne Diseases, Department of Disease Control, Ministry of Public Health, Nonthaburi, Thailand; 3 World Health Organization, Mekong Malaria Programme, c/o Faculty of Tropical Medicine, Mahidol University, 420/6 Rajvithi Rd, Bangkok, 10400, Thailand; 4 Department of Tropical Hygiene, Faculty of Tropical Medicine, Mahidol University, Bangkok, Thailand

**Keywords:** Malaria containment, Electronic-based, Information system, Malaria surveillance

## Abstract

**Background:**

The Bureau of Vector-borne Diseases, Ministry of Public Health, Thailand, has implemented an electronic Malaria Information System (eMIS) as part of a strategy to contain artemisinin resistance. The attempt corresponds to the WHO initiative, funded by the Bill & Melinda Gates Foundation, to contain anti-malarial drug resistance in Southeast Asia. The main objective of this study was to demonstrate the eMIS’ functionality and outputs after implementation for use in the Thailand artemisinin-resistance containment project.

**Methods:**

The eMIS had been functioning since 2009 in seven Thai-Cambodian border provinces. The eMIS has covered 61 malaria posts/clinics, 27 Vector-borne Disease Units covering 12,508 hamlets at risk of malaria infections. The eMIS was designed as an evidence-based and near real-time system to capture data for early case detection, intensive case investigation, monitoring drug compliance and on/off-site tracking of malarial patients, as well as collecting data indicating potential drug resistance among patients. Data captured by the eMIS in 2008–2011 were extracted and presented.

**Results:**

The core functionalities of the eMIS have been utilized by malaria staff at all levels, from local operational units to ministerial management. The eMIS case detection module suggested decreasing trends during 2009–2011; the number of malaria cases detected in the project areas over the years studied were 3818, 2695, and 2566, with sero-positive rates of 1.24, 0.98, and 1.16%, respectively. The eMIS case investigation module revealed different trends in weekly *Plasmodium falciparum* case numbers, when classified by responsible operational unit, local and migrant status, and case-detection type. It was shown that most Thai patients were infected within their own residential district, while migrants were infected either at their working village or from across the border. The data mapped in the system suggested that *P. falciparum*-infected cases and potential drug-resistant cases were scattered mostly along the border villages. The mobile technology application has detected different follow-up rates, with particularly low rates among seasonal and cross-border migrants.

**Conclusion:**

The eMIS demonstrated that it could capture essential data from individual malaria cases at local operational units, while effectively being used for situation and trend analysis at upper-management levels. The system provides evidence-based information that could contribute to the control and containment of resistant parasites. Currently, the eMIS is expanding beyond the Thai-Cambodian project areas to the provinces that lie along the Thai-Myanmar border.

## Background

During the last decades, the Greater Mekong Subregion (GMS) has been experiencing the highest level of *Plasmodium falciparum* resistance to anti-malarial medicines (as monotherapies or in combination) in the world. More recently, evidence of growing *P. falciparum* resistance to artemisinin and its derivatives has been reported along the Thai-Cambodian border [1,2]. Artemisinin-based combination therapy (ACT) is the most rapid, reliable and effective treatments to cure patients infected with *P. falciparum* malaria worldwide. In addition to possibly compromising results in the Asia-Pacific region, growing falciparum resistance to artemisinin derivatives was considered to be a global public health threat, possibly affecting global efforts and investments if no action is taken, with the potential for particularly ill effects in Africa [3]. Moreover, only a few replacement therapies in the latter phases of drug development are in the pipeline. With the serious concern that resistance may spread beyond the Greater Mekong Subregion to affect other regions, the World Health Organization (WHO) has developed and supported a bi-country and multi-partner initiative to contain artemisinin-resistant parasites. The initiative was later consolidated into a global plan for artemisinin-resistance containment in 2011[4]. The multi-pronged containment strategy was based on several elements: (1) stop the survival and spread of resistant parasites, (2) increase monitoring and surveillance to identify new foci rapidly, and provide information for containment and prevention activities, (3) increase access to diagnostics and treatment with ACT to improve patient outcomes and limit opportunities for resistance, (4) invest in anti-malarial drug resistance-related research, and (5) motivate stakeholders at global, regional and national levels to support containment activities [4].

One key objective of the containment operation was to strengthen programme management through close observation and effective coordination with partners, which would enable rapid and high-quality strategy implementation. The main activities were laid out to ensure that all suspected patients had access to reliable diagnostic tools in the target areas, and to ensure all infected patients had access to and use of radical treatments, including gametocidal drugs [5,6].

Thailand’s Bureau of Vector-borne Diseases (BVBD) is vertically driven in the border provinces that remain endemic for malaria. This means that special malaria services are operating in all endemic and remote villages in concert with general healthcare services. At the most peripheral points-of-care, such malaria posts and clinics, and in malaria-endemic communities, village malaria volunteers and/or village health volunteers work in collaboration with malaria program staff. At the upper levels, treatment and care activities are monitored vertically by the Vector-borne Disease Unit, Vector-borne Disease Center, Regional Offices of Disease Prevention and Control, and the BVBD. In addition to passive case detection, in which local febrile patients visit a malaria post/clinic or Vector-borne Disease Unit office for diagnosis by themselves, the local operational staff also conduct periodic active case detection in the villages for which they are responsible.

As part of the containment project implemented in seven provinces along the Thai-Cambodian border, the electronic Malaria Information System (eMIS) was developed based on the aforementioned existing malaria-prevention and -control activities. The eMIS was built on the original hand-processed paper-based system that has not been significantly altered by Thailand’s National Malaria Prevention and Control Programme in the past 5 decades. The paper-based system was time-consuming, slow in sharing and consolidating data across peripheral units, and did not provide consistent information at the central level for timely decision-making and reporting to the highest political level.

Even though the eMIS was specifically developed to address the problem of artemisinin resistance, it was also designed eventually to replace the BVBD’s existing paper-based system, to reduce redundant data across routine data-collection forms and different aggregate data reports, whilst enhancing systems with modern technology and communication features. Replacing paper-based and aggregated data flow with integrated web and mobile technology was expected to provide real-time, traceable evidence at all levels for case detection at the point-of-care, case investigation, and follow-up activities for individual cases. The information within the system was expected to be used at both operational and supervision levels for case management, individual case follow-up and intervention coverage purposes. The primary objective of the study was to demonstrate the design and implementation of eMIS’s functionalities. The secondary objective was to present the preliminary results of the system after use as an active, evidence-based malaria surveillance system for the Thailand malaria-containment project. The project’s main containment outcome regarding parasite clearance is discussed in the second part of this manuscript series.

## Methods

### Implementation locations

**Figure 1:**
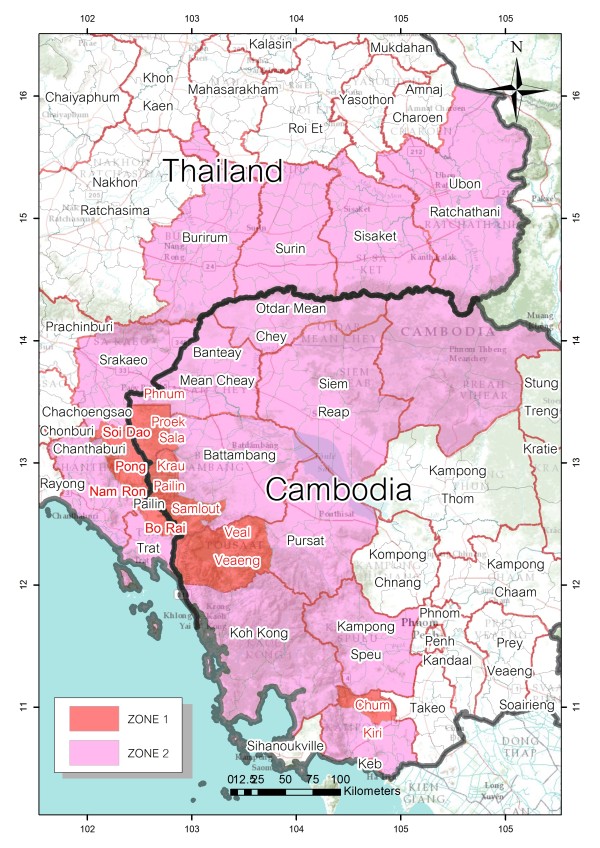
Classification of zones in the Thai-Cambodian malaria-containment project.

For the purpose of incorporating the new system into the mainstream program, the eMIS was developed by the Center of Excellence for Biomedical and Public Health Informatics (BIOPHICS) in close collaboration with the National Malaria Programme managed by the BVBD. The eMIS was initially piloted in endemic zones of two Thailand provinces along the Cambodian border, where artemisinin resistance was documented as highest (designated as zone 1 in the project). The BVBD, however, decided to implement the system progressively in all seven Thai provinces bordering Cambodia, where, at that time, there was no evidence of artemisinin resistance (designated as zone 2 in the project). Thus, the eMIS was functioning only on the Thailand side of this bi-country containment project. Figure 1 is a map of the project’s containment areas on both sides of the Thai-Cambodian border. At full scale, the eMIS covered 61 malaria posts/clinics, 27 Vector-borne Disease Units, 11,615 villages, and 12,508 hamlets.

### Work and data flow

The eMIS was primarily designed to digitize malaria data generated from peripheral passive case-detection units and from intensive index-case investigations, to monitor anti-malarial drug compliance, supervise on/off-site tracking of malarial patients under treatment, to ensure strict follow-up, and to detect/report real-time any inadequate clinical or parasitological response to the drug. In addition, disease mapping and spatial analysis were incorporated into the system to increase the performance of rapid response teams in implementation areas. Moreover, although the eMIS was specifically developed to contain *P. falciparum* cases, it also records infection information on all other species, and the case-management routines and work activities of malaria-control programmes.

The eMIS combines web-based and mobile technology to achieve its functionalities. The four main modules of the web-based system include: (a) case registration, (b) case investigation, (c) case follow-up, and (d) VIVO laboratory results. Google Earth was built into eMIS so that diseases can be mapped almost real-time. Maps portraying malaria situations can be customized by local operational staff at each level to present information at village and household levels. The mobile technology function employs “smart-phones” for data capture and alert messages.

**Figure 2:**
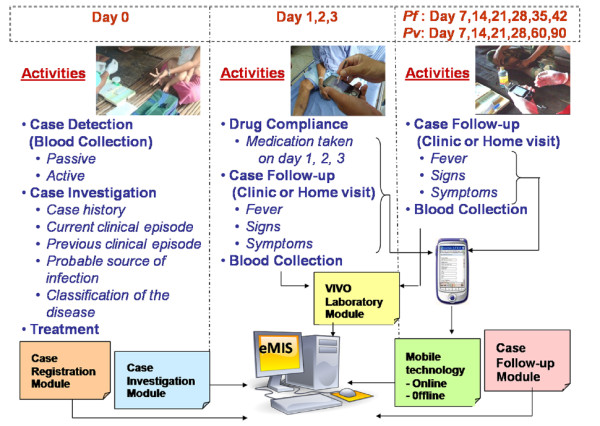
Work and data flow of the Thailand malaria-containment project.

In the case-registration module, the data captured for passive and active case detection (including basic demographics) of both infected and non-infected cases were entered into the eMIS. Details of case investigations and treatments provided, as shown in Figure 2, were captured for all documented infected cases. In addition, particularly for *P. falciparum*-infected cases, attempts have been made to capture and assess drug compliance on days 1, 2, and 3. Case *P. falciparum* and *Plasmodium vivax* cases were followed up routinely, to record temperature, symptoms, and blood-draw, for monitoring treatment outcomes and parasite clearance. Blood smears were collected at registration and during clinic or home follow-up visits for microscopic and PCR analysis; however, not all smears were analysed by PCR or utilized for routine malaria surveillance.

### Ethical considerations

eMIS access and operational modules were integrated into the routine work performed by malaria and public-health personnel. All patient-management activities, and the database containing information associated with the described activities above, which formed part of the eMIS, utilized strict security and were used only by authorized personnel in charge of patient case-management. Similar to data captured within the original paper-based reporting system, the eMIS has maintained the same crucial data integrity and confidentiality. No written informed consent forms were signed by patients who visited the malaria clinics, since the activities were considered routine programmatic malaria procedures and interventions. However, the malaria staff informed all patients verbally and asked them to return to the clinic or agree to home visits, to be conducted as part of their scheduled 28-day follow-up. Data extracted from the eMIS database were secondary data with no identification linked to any individual patient. The authors requested official permission from the Ministry of Public Health (Director of the BVBD) to extract these unlinked digital data for further analysis as part of the joint BIOPHICS-MOPH collaboration. The protocol for this methodology was reviewed and approved by the Ethics Committee of the Faculty of Tropical Medicine, Mahidol University.

## Results

Development of the eMIS began in January 2009, and the program was launched in July 2009. At the end of December 2011, over 800,000 records were entered into the system. As part of the requirements of the artemisinin resistance-containment strategy to ensure an “effective management, surveillance and coordination” system, the following eMIS functionalities and outputs have been developed and scaled up for use in the field.

### Reporting system

Since the eMIS was meant to be used as part of the routine programmatic work of the national malaria-prevention and -control programme, its core functionalities purposively maintained the original vertical reporting system whilst adding specific data-entry procedures for entering individual case detection and investigation information directly into the system. The follow-up information recorded during the home visit was captured online and offline by smart phones provided to malaria and healthcare staff. The data were then transmitted automatically to the system, or synchronized later if cell-phone use was not possible in a follow-up area. With near real-time entry of data and transmission for individual case deection and investigation (from day 0) case follow-up visits into the system, authorized staff at all levels, from local units to BVBD, had the opportunity to gather, review and critically assess the malaria situation in their own areas of responsibility and elsewhere each day. These features and automatic procedures made it much simpler to aggregate data from different types of reports and from different operational units up to the highest level of decision-making. Routine reports of aggregated data with traceable records were able to be generated whenever required. For purposes of data-quality control, especially in the early phase of implementation, the malaria staff maintained, cross-checked, and reconciled the eMIS data captured with those collected from the original paper-based system. The data-completion rates and data quality of the paper-based mechanism were compared with the eMIS, and presented at meetings of management-level malaria-control personnel; there was evidence of incomplete and missing data, as well as data inconsistencies, but the situation improved as the staff became more experienced. It should be noted that, in the early phase of eMIS implementation, data collection and reporting from both routine paper-based and implementation-phase electronic-based systems were done by trained malaria personnel working for the existing national vertical malaria-control programme. The paper-based mechanisms were then progressively phased out in the project areas.

**Figure 3:**
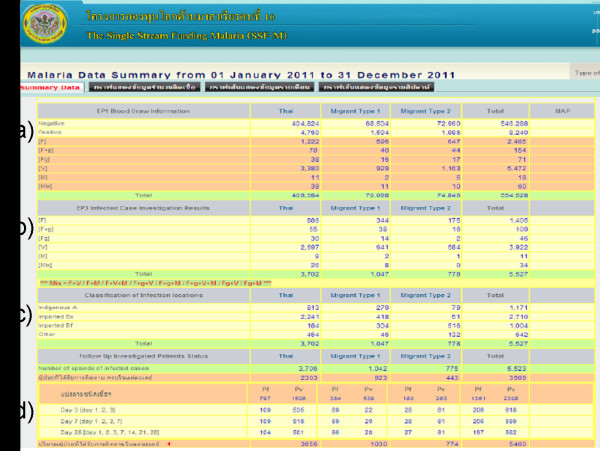
**Screenshot of eMIS public access page (www.biophics.org/malariar10) presenting summary statistics of malaria cases along the Thai-Cambodian border, classified by nationality (Thai, Long-term migrant type 1, Seasonal and cross-border migrant type 2, and Total).** (**a**) Total figures for blood drawn, organized by microscopic test results and species of infection; (**b**) Total numbers of infected cases investigated, by species type; (**c**) Classification of self-reported infection locations – indigenous A, imported Bx, imported Bf, other; (**d**) Complete follow-ups at Day 3, 7, and 28.

**Figure 4:**
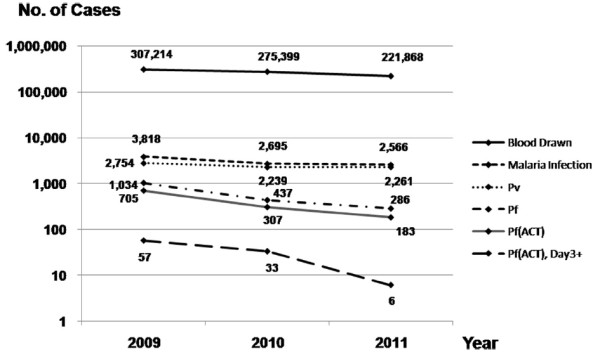
Summary statistics of malaria cases in 7 provinces along the Thai-Cambodian border, 2009–2011.

Figure 3 shows a screenshot of a public-access page that summarizes data from malaria cases collected to the present day (http://www.biophics.org/malariar10). Linking to this webpage, the most up-to-date statistics are presented on public pages, but some pages are restricted to authorized BVBD personnel. The summary of cases digitized in the eMIS database, between January 2009-December 2011, are shown in Table 1 and Figure 4. Even though the eMIS was officially launched in mid 2009, original paper-based data collected prior to the eMIS’ launch were entered retroactively into the electronic system. Consolidated tables and graphs displayed by day, week, month, or year show a decreasing trend of confirmed malaria cases over the past three years in the operational project area. As shown in Table 1, the overall slide-positive rate (all malaria species) was 1.24% (3,818/307,214), 0.98% (2,695/275,399) and 1.16% (2,566/221,868), in 2009, 2010, and 2011, respectively. As expected, the proportion of *P. vivax*-infected patients among the confirmed cases increased from 72% to 88% with more recorded positive cases among migrants in zone 1 than zone 2. It should be noted that not all cases were treated by ACT; as shown in Table 1, the percentages of cases receiving ACT treatment were 68, 70, and 64%, during the period 2009–2011, respectively. The malaria statistics during the three years (2009–2011), summarized in Figure 4, represent cumulative annual statistics captured on the eMIS public-access page.

**Table 1:** Summary of malaria cases in 7 provinces along the Thai-Cambodian border (January 2009-December 2011)

	**2009**		**2010**		**2011**	
	**N**	**%**	**N**	**%**	**N**	**%**
Total blood draw	307,214		275,399		221,868	
Passive case detection (PCD)	92,846	30	80,608	29	63,906	29
Thailand	86,184	93	75,660	94	59,236	93
Cambodia	5,237	6	3,686	5	3,928	6
Myanmar	387	0	238	0	255	0
Other	1,038	1	1,024	1	487	1
Active case detection (ACD)	214,368	70	194,791	71	157,962	71
Thailand	137,155	64	115,853	59	87,124	55
Cambodia	59,026	28	63,129	32	58,402	37
Myanmar	15,491	7	12,640	6	9,291	6
Other	2,696	1	3,169	2	3,145	2
Malaria Infections	3,818	1.24*	2,695	0.98*	2,566	1.16*
*P. vivax*	2,754	72	2,239	83	2,261	88
*P. falciparum*	1,034	27	437	16	286	11
- Zone 1	73	7	37	8	27	9
Thai	49	67	23	62	12	44
M1	8	11	7	19	4	15
M2	16	22	7	19	11	41
- Zone 2	961	93	400	92	259	91
Thai	868	90	350	88	222	86
M1	44	5	16	4	8	3
M2	49	5	34	9	29	11
*P. falciparum* treated with ACT)	705	68	307	70	183	64
*P. falciparum* treated with ACT remaining positive on Day 3	57	8	33	11	6	3

* Slide positive rate (SPR) based on malaria infection divided by total blood draw.

### Situation and trend analysis

The eMIS offered customized motion graphs and reports, which could be generated based on near-real-time digitized databases. It allowed authorized malaria staff to access, and assess, the malaria situation and trends anywhere in the project area in a timely manner. Graphs and reports can be specifically customized according to the skills and needs of field personnel, location and time. Peripheral operational units and upper management levels can get access to data and generate reports within their BVBD assigned roles and responsibilities. This feature assisted supervising malaria staff in acting upon occurring cases in their localities of responsibility, strengthening the performance of individual case management (under Direct Observed Treatment, DOT) and individual follow-up by allowing staff to take remedial measures and appropriate action in a timely manner.

**Figure 5:**
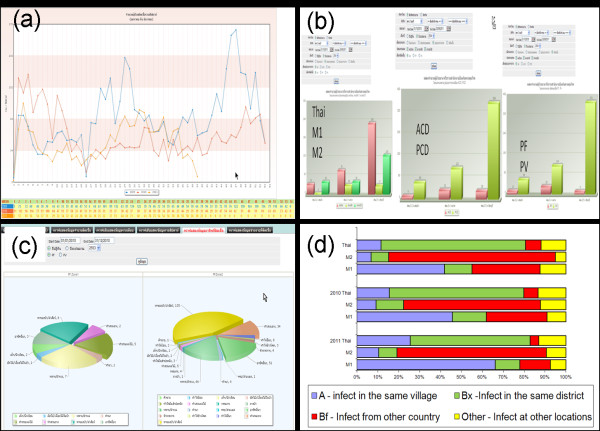
**Examples of situation and trend analyses as part of the eMIS presentations.** (**a**) Motion line graph of weekly numbers of cases, January 2009-August 2011; (**b**) Bar graph of cases with different characteristics by selected operational unit; (**c**) Pie chart of cases among different occupations at risk in selected provinces; (**d**) Bar graph of case investigations on self-reported locations of infection.

Figure 5 shows some examples of system-generated graphs, which help staff at operational units and/or upper management at ministerial levels to estimate the current malaria situation and trends. As shown in Figure 5 (a), trends of *P. falciparum* cases over the past three years (2009–2011) reveal that the overall number of malaria cases appears to decrease over weeks, but fluctuations are noted in certain periods. This prompted the management level to alert the operational units to monitor the situation carefully and act accordingly. Figure 5 (b) shows bar graphs generated for different purposes, e.g., cases in different operational units classified by nationality, parasite infection and methods of detection. Caseloads are different among operational units pertaining to the citizenship of patients (Thais, long-term Migrant type 1 (M1 = staying in Thailand ≥ 6 months), and short-term/seasonal Migrant type 2 (M2 = staying in Thailand < 6 months and/or mobile cross-border population)). The number of infected patients detected by active case detection (ACD) was much less than those detected by passive case detection (PCD), reflecting some difficulty in performing ACD in certain remote localities. Over several years, the number of *P. vivax*-infected cases has been increasing across all operational units. Figure 5 (c) reveals different occupational risks in different border province. As seen in the example of two provinces, there was high percentage of infection among soldiers in certain province directly affected by border conflict, while a similarly high percentage was noticed among cross-border farm/orchard workers in another province. Figure 5 (d) indicates that most Thai patients were infected within the district, while M1s were infected in their working village, and M2s were likely infected from abroad.

### Geographical Information System (GIS) applications

**Figure 6:**
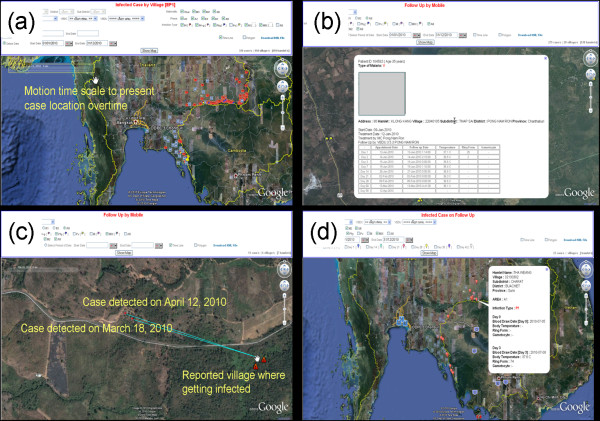
**Examples of temporal and spatial presentations of malaria cases within the eMIS-GIS functionality.** (**a**) *P. falciparum* cases mapped at village level with motion timescale to present locations of cases occurring during the selected time frame; (**b**) *P. falciparum* cases mapped at household level with follow-up details of a selected case’s demographics, temperature, and parasite/gametocyte data; (**c**) Self-reported locations of infection, based on case investigations; (**d**) Day 3 locations of positive cases on a map, with details of a selected case on follow-up parasite data.

Employing GIS technology, the motion spatial and temporal presentations within eMIS were utilized by staff to identify and locate follow-up cases, and to assess temporal and spatial situations within their areas of responsibility. Figure 6 shows some examples of GIS presentations in the system. As shown in Figure 6 (a), *P. falciparum* -infected cases (Thais and non-Thais) in the seven provinces were scattered mostly along the border villages. There were more absolute cases in the northeastern provinces, probably due to the larger population size. Figure 6 (b) shows cases mapped at the household level, where local malaria staff performed home visits. Information acquired from individual follow-ups can be displayed for each case investigated. The GIS could also be used by malaria health staff to identify areas targeted for home visits. Dates of appointment were automatically generated to process individual case investigations and to plan subsequent control measures if needed. From self-reported information gathered during case investigations, the location where patients were most likely infected was mapped, as shown in Figure 6(c). This feature had the potential to update prevention and control measures in problematic locations. It should be noted, however, that the potential location of infection was based on self-reporting during atypical case investigation processes.

One of the main objectives of using the eMIS in the containment project was to map patients still positive after 3 days’ ACT treatment. Mapping locations with suspected *P. falciparum* resistance to artemisinins in near-real-time allowed the containment project to perform case investigation instantly, to detect and treat secondary cases radically. Day 3 positivity was recorded utilizing routine surveillance data collection (simply counting days post-treatment, without details, such as hour of treatment) during case follow-up, either at a malaria clinic or through home visits by malaria personnel. Even though day-3 parasitaemia is an insensitive marker of artemisinin resistance, it is the best available indicator used by the project to routinely measure *P. falciparum* sensitivity to artemisinins [7]. Therefore, such an indicator on the map could help by screening for potential artemisinin resistance. Figure 6 (d) depicts GIS mapping of such cases at the village level for artemisinin resistance containment.

### Mobile technology applications and alert system

**Figure 7:**
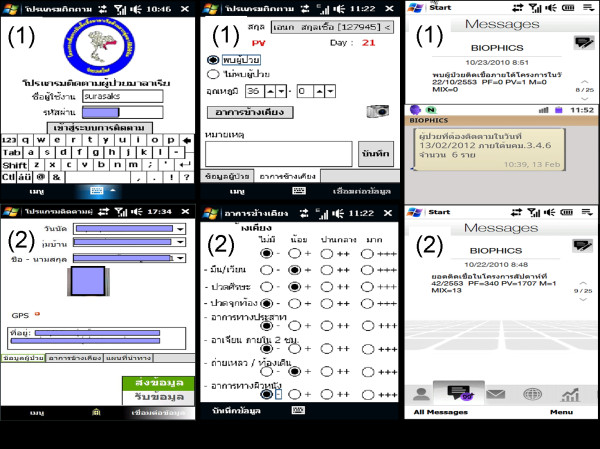
**Screenshots of mobile-phone applications and SMS alert system.** (**a-1**) Login screen of the password-secured mobile phone system; (**a-2**) Infected-case details with patient name and picture (as per permission), appointment date, address to be submitted online or offline during case investigation or home visit follow-up; (**b-1**) Case follow-up details on temperature, side effects, and other remarks; (**b-2**) Check boxes of atypical malaria signs and symptoms captured during home visit follow-ups; (**c-1**) Daily SMS alert messages – Day 0 alert informing number of cases within the specific malaria clinic area of responsibility, and a case follow-up alert informing number of cases to be followed up on a particular day; (**c-2**) Weekly SMS alert messages informing of the cumulative number of cases, particularly for each level of the vertical malaria control programme, from local operational units to ministerial management level.

As part of the eMIS, an application running on a Windows-based mobile phone was developed to record patient information. The mobile device was selected for easy portability to the patient’s location during home visits, to store collected data and send the data either immediately online or once signal coverage became available. The mobile-phone applications developed for case follow-up at offsite locations in remote areas captured text, images, and locations. Figure 7 (a) and (b) shows that, during case follow-up activities outside a malaria clinic, the staff collected a blood specimen to monitor treatment outcomes while capturing information on the mobile phone to assess drug compliance, clinical signs (if any), and patient locations.

Mobile technology applications were also used to track patients and remind staff about specific follow-up activities. As soon as an infected patient was registered in the eMIS database, a follow-up schedule was automatically generated, by malaria species. On the scheduled date, the system reminded the staff responsible to perform an individual case follow-up on the mobile phone provided; this functionality was particularly useful to ensure home visits in remote areas. Efforts were made to perform follow-up visits for all scheduled dates; however, due to difficulties in coordinating case follow-up, particularly for cross-border and/or unregistered migrant workers, many scheduled follow-ups had to be skipped. Moreover, it was not standard practice to conduct frequent follow-up visits across malaria-endemic zones in all seven provinces, which was made evident by the fact that few or no visits were carried out in some areas. As shown in Tables 2, the overall follow-up rate (compared with scheduled visits) varied by location, between 50-90% for both *P. falciparum* and *P. vivax* cases. The follow-up rate was slightly higher among Thai citizens than migrants, with an exception in 2011, when the follow-up rate was higher among migrants than Thais. Even though the follow-up was scheduled until day 42 for *P. falciparum* cases and day 90 for *P. vivax* cases, it remained high at day 42 and day 90 (80% & 70%). However, during several off-site follow-up visits, most notably in remote areas, some specimen collections for laboratory testing were not performed.

**Table 2:** **Summary of*P. falciparum*-case and*P. vivax*-case follow-up in 7 provinces along the Thai-Cambodian border (January 2009-December 2011)**

**P. falciparum**									
**Follow-up Days**	**2009**			**2010**			**2011**		
	**Thai**	**M1**	**M2**	**Thai**	**M1**	**M2**	**Thai**	**M1**	**M2**
	**N (%)**	**N (%)**	**N (%)**	**N (%)**	**N (%)**	**N (%)**	**N (%)**	**N (%)**	**N (%)**
Day 0	911	50	65	363	23	39	214	11	33
Day 1	590 (65)	25 (50)	13 (20)	235 (65)	15 (65)	20 (51)	69 (32)	9 (82)	20 (61)
Day 2	589 (65)	25 (50)	13 (20)	236 (65)	16 (70)	20 (51)	69 (32)	9 (82)	20 (61)
Day 3	589 (65)	25 (50)	13 (20)	234 (64)	17 (74)	20 (51)	69 (32)	9 (82)	20 (61)
Day 7	741 (81)	46 (92)	31 (48)	322 (89)	18 (78)	25 (64)	158 (74)	11 (100)	30 (91)
Day14	584 (64)	26 (52)	13 (20)	226 (62)	14 (61)	20 (51)	68 (32)	9 (82)	20 (61)
Day 21	581 (64)	26 (52)	13 (20)	223 (61)	15 (65)	20 (51)	67 (31)	8 (73)	20 (61)
Day 28	727 (80)	45 (90)	31 (48)	309 (85)	15 (65)	25 (64)	156 (73)	10 (91)	29 (88)
Day 35	702 (77)	45 (90)	31 (48)	300 (83)	13 (57)	25 (64)	151 (71)	9 (82)	27 (82)
Day 42	707 (78)	45 (90)	31 (48)	296 (82)	13 (57)	25 (64)	150 (70)	9 (82)	26 (79)
P. vivax									
**Follow-up Days**	**2009**			**2010**			**2011**		
	**Thai**	**M1**	**M2**	**Thai**	**M1**	**M2**	**Thai**	**M1**	**M2**
	**N (%)**	**N (%)**	**N (%)**	**N (%)**	**N (%)**	**N (%)**	**N (%)**	**N (%)**	**N (%)**
Day 0	2063	266	412	1685	163	389	1850	111	378
Day 1	925 (45)	89 (33)	115 (28)	873 (52)	94 (58)	259 (67)	599 (32)	23 (21)	61 (16)
Day 2	923 (45)	89 (33)	116 (28)	877 (52)	89 (55)	261 (67)	591 (32)	23 (32)	61 (16)
Day 3	918 (44)	89 (33)	115 (28)	875 (52)	88 (54)	262 (67)	585 (32)	23 (21)	61 (16)
Day 7	919 (45)	89 (33)	115 (28)	870 (52)	89 (55)	261 (67)	581 (31)	22 (20)	60 (16)
Day14	1585 (77)	137 (52)	280 (68)	1315 (78)	96 (59)	305 (78)	1142 (78)	83 (75)	264 (70)
Day 21	920 (45)	89 (33)	114 (28)	839 (50)	83 (51)	254 (65)	557 (30)	23 (21)	55 (15)
Day 28	1571 (76)	139 (52)	279 (68)	1316 (78)	91 (56)	298 (77)	1288 (70)	87 (78)	257 (68)
Day 60	1536 (74)	132 (50)	279 (68)	1194 (71)	70 (43)	282 (72)	1121 (61)	69 (62)	198 (52)
Day 90	1533 (74)	131 (49)	279 (68)	1167 (69)	72 (44)	278 (71)	1240 (67)	54 (49)	177 (47)

The Short Messaging Service (SMS) was incorporated into the eMIS as an alert system. As shown in Figure 7 (c), daily new infected case(s), as well as cumulative positive case alerts, were set up to inform malaria staff at local and upper levels about the current malaria situation in their areas of responsibility. Daily infections and a weekly summary of all malaria cases were automatically sent to the head of each Vector-borne Disease Unit for the purpose of broadcasting the malaria situation in a given period of time. The daily and weekly messaging feature helps alert staff at the local level to manage each single case, and assess the local situation on a quasi-real-time basis.

## Discussion

The eMIS has been evolving over several years with continuous input from end-users, who have provided useful feedback to BIOPHICS to assist in matching the peripheral technical requirements and constraints of local units with the programmatic needs of regional and central management offices. Despite different needs and requests across working units in different malaria-endemic areas of the seven provinces, the system eventually incorporated all concerns with standardized operational practices to generate data across units, therefore reducing double-entries and repetitiveness, and reducing time-consuming effort whilst generating timely, quality cross-checked reports.

During the early phase of system implementation, it took some time for staff to learn to use the system effectively. Several issues arose regarding the management of the hardware and the undeveloped skills of existing staff. While the electronic system evolved, there were physical and psychological effects on staff due to the additional workload as the result of two reporting systems running in parallel (paper- and electronic-based). Several training sessions coupled with progressive improvements to the eMIS, with additional features, increased end-user interest in the new system. This, in turn, allowed BIOPHICS to receive better feedback from, and collaborate more efficiently with, peripheral health staff. Over time, the upper management level at BVBD was also convinced of the added value of a system created to monitor and assess progress made through critical containment interventions with quick remedial actions in their areas of responsibility.

Contributions by the eMIS improved the ability of malaria-surveillance systems to capture data daily from individual malaria patients, almost eliminating the need to collect aggregated monthly data from local operational units. It should be noted, however, that some discrepancies were identified between the original paper-based system and the new electronic-based system in the data reported to upper levels, especially at the beginning of the project when the two systems were still running in parallel. Such findings have been explained as double counting certain papers, data not being digitized at all, or data wrongly introduced to the database. These issues have been progressively addressed by all parties. When cross-checked, consolidated numbers from the eMIS eventually represented the figures reported by the Bureau of Vector-borne Diseases (BVBD), which may differ slightly from the statistics provided by the Bureau of Epidemiology (BOE), which gathers data from additional health sources, such as hospitals. Even though sharing information is a routine practice between the two authorities, with almost all malaria cases being managed free-of-charge by official health facilities (off-the-shelf treatments for malaria are not permitted in Thailand), some patients go directly to official health facilities, clinics, and even hospitals, so they do not get counted on paper, or electronically. This has been an issue with malaria reporting in Thailand over the decades; however, in recent years, the numbers of the two reporting mechanisms have been quite close. It is predicted that a functioning eMIS, which encompasses all healthcare facilities, not just malaria, will further address that issue in the next version of the eMIS.

Individual data recorded in the eMIS can be exported in different formats for further epidemiological analysis. Raw data extracted from the eMIS can then be used to generate reports for authorized staff. Information gathered by the eMIS in 2009–2011 indicated a decline in slide-positivity rate in the 7 provinces from 1.24 to 1.16%. This may be due in part to the intensive early-case-detection and case-management efforts in the containment project. The statistical data collected electronically from each village in each specific jurisdiction, coupled with reclassification for different types of malaria endemicity, make calculating risk by village easier than using the paper-based system.

Several other issues regarding the system implementation require attention. Operational practices were not consistent between different operational units in terms of detection methods, individual follow-up, recording citizenship, occupation, etc. The issues of data integrity and standardization have been discussed elsewhere [8-12]. The collection of more detailed evidence from each operational unit will inform the redesign or fine-tuning of locally-driven prevention and control measures. More comprehensive information will also assist in reallocating resources and efforts to address rapidly evolving situations for each operational unit, which differ from generic, more static measures that are unlikely to deal with disease elimination. The mobile migrant population remained the major concern for the malaria-prevention and -control programme. The system highlighted the high percentage of mobile workers who could not be followed up by malaria staff in the Cambodian border containment area. The case follow-up rate for migrants in the Thai-Cambodian containment area was rather low (as low as 20%) compared with the findings of the Microsoft Research-funded study [13] piloted in one district on the Thai-Myanmar border, where the malaria follow-up rate was > 80%. Even though the eMIS had similar mobile technology and follow-up module to assist patient tracking as the Microsoft study, the results were different, because most migrants in the latter study were long-term residents of Thailand, and not highly mobile. However, the containment project was implemented in all order districts on the Thai-Cambodian border, where short-stay seasonal migrant workers are numerous; in addition, these areas included migrants from Cambodia seeking healthcare in Thailand. Ensuring that all mobile people, whatever their citizenship, have access to treatment and preventive measures is one of the main containment strategies [14-17]. Therefore, to eliminate artemisinin-resistant strains, more innovative strategies, and the involvement of more key stakeholders (e.g., national and international NGOs working on migrant issues) should be considered. The low follow-up rate among cross-migrant workers along the border could be improved by malaria staff and/or NGOs collaborating with the migrants’ managers or orchard owners. In highly endemic areas, out-of-normal-hour service in malaria clinics may improve minimum required follow-up visits.

Despite these issues, the eMIS’ features, combined with coordinated supervision, have demonstrated the capacity to provide malaria staff and the MOPH/BVBD with quality, quasi-real-time information, allowing them to make accurate decisions on disease control and planning in target areas. The BVBD, backed by BIOPHICS, took advantage of the eMIS system better to monitor the malaria situation in peripheral areas, and could alert local staff to act upon events in a timely manner. The inter-connected modules within the system have shown that the system contributed to an improvement in case management and individual follow up, which in turn helped to improve real-time situation assessment and epidemiological knowledge, and helping to identify the determinants of malaria spread. It also helped malaria workers to identify and track potential resistant parasites more effectively. The outcomes from the eMIS were quite positive, and could potentially be adopted and supported in the national policy to eliminate malaria in Thailand.

The outputs of the eMIS were similar to other information systems developed for malaria prevention and control. The quality of the system’s data has enabled malaria personnel to perform their duties better. The systems developed elsewhere [18-20] were implemented in specific settings, and it was suggested that these systems could be integrated into national malaria control programmes at Ministry level. The eMIS, however, was implemented as part of the routine regional malaria-control programme. This approach was initially planned in the design phase of the eMIS, to assure the robustness and sustainability of the system should it prove effective in malaria-case management and become well-accepted for use as the official malaria information system.

Several GIS applications were developed for case surveillance and vector control (coverage by community-based spraying, insecticide consumption and application rates) and preventive measures (coverage of insecticide-treated nets) [21-24]. The information communication technology (ICT) used in malaria control in Tanzania [20] suggested ICT could result in easier communication, improved training for doctors, and increased access to information by individuals and groups who are historically unaware of malaria. These information system functionalities are planned for the next version of the eMIS.

A recent review of the research agenda for the monitoring, evaluation and surveillance of malaria, with the end-goal of eradication [3], suggested that surveillance technologies based on cell-phone or real-time internet web-based reporting, be evaluated, since these new strategies could have major implications for program implementation. An assessment of any delivery system should cover acceptability, feasibility, efficiency, cost-effectiveness, and community engagement. There has been no formal evaluation of the eMIS; however, the system has been imbedded into routine work and appears to have been accepted by system users (based on informal interviews). The main system costs comprised the acquisition and maintenance of system hardware (computer systems and mobile phones), and the employment of new ICT personnel at some operational units. These costs were supported by the containment project initiative under the management of BVBD. However, the project has not yet been evaluated for cost-effectiveness.

## Conclusion

The eMIS was developed and employed in provinces where intensive containment operations took place, to increase the overall performance of the malaria surveillance system, to strengthen case-management and strict follow-up of all malaria patients, and to improve the coordination of data management between local malaria operational units and upper programmatic management levels. The outputs from the eMIS have demonstrated that the system provides almost-real-time evidence-based information on individual malaria cases managed in local operation units, and can be used effectively for situation and trend analysis by senior management. The eMIS is an improvement on the paper-based system, where the information was aggregated vertically and submitted routinely. This timely information can contribute to more effective case management and thus to the coordinated control and containment of resistant parasites.

Uptake for routine malaria case management in all seven provinces along the Thai-Cambodia border took some time, and the development of a public-health informatics system is ongoing. Many additional features were developed during the project. Lessons learnt from the project were (1) cooperation and support by central management is key to eliminating resistant parasites; (2) program sustainability is required for continued system enhancement, and (3) participation and feedback from all operational levels can assist to generate viable results and improve performance.

Recently, the Global Fund Round 10 [25] has provided support for Thailand’s expansion of containment efforts across its regional borders; the eMIS is now expanding into provinces along the Thai-Myanmar border. The limitations and drawbacks of the system revealed during the containment project along the Thai-Cambodian border are being resolved. Discrepancies between paper-based and electronic reports, and different channels of reporting between the two disease control authorities, are being evaluated. It is anticipated that the paper-based system of malaria surveillance will be phased out as the project progresses. As part of the Global Fund Round 10 proposal, BIOPHICS’ developers are designing additional prevention and control modules, including entomology data, quality control of microscopic-test data, behavioral-communication-change modules, and bednet/insecticide residual spraying (IRS) distribution. When all prevention and control modules are combined, it is expected that the new informatics tool will fully support vector-borne disease-control operations, and provide an intelligent choice for assisting containment activities. This is an opportunity to prove that new information technology can be used as a tool for disease prevention and control, as well as an effective communication channel to the population at risk. It is anticipated that the enhanced eMIS could be applicable for Thailand at large, and other countries in the region, and beyond.

## Competing interests

The authors declare that they have no competing interests. eMIS development was supported by the malaria containment initiatives of the WHO and the Bill & Melinda Gates Foundation.

## Authors’ contributions

AK, JK, PS(1) were involved in the conceptualization and design of the study. SS, TP, AS designed the system and its applications. SS, TP, AS designed and programmed each application module, monitored and maintained the module’s implementation, and extracted data for analysis. PS(1), WS, SV were responsible for managing and supervising the overall malaria-control-programme activities. AK, JK, and DC were in charge of monitoring the progress of the module application. AK, JK, PS(1), SL, PS(2) performed statistical analyses and drafted the manuscript. All authors read and approved the final manuscript.

## References

[B1] World Health Organization Containment of malaria multi-drug resistance on the Cambodia-Thailand border 2007 Report of an Informal Consultation Phnom Penh, Cambodia

[B2] Dondorp AM Nosten F Yi P Das D Phyo AP Tarning J Lwin KM Ariey F Hanpithakpong W Lee SJ Ringwald P Silamut K Imwong M Chotivanich K Lim P Herdman T An SS Yeung S Singhasivanon P Day NP Lindegardh N Socheat D White NJ Artemisinin resistance in Plasmodium falciparum malaria NEJM 2009 361 455 467 10.1056/NEJMoa0808859 19641202 PMC3495232

[B3] Anderson TJ Roper C The origins and spread of antimalarial drug resistance: lessons for policy makers Acta Trop 2005 94 269 280 10.1016/j.actatropica.2005.04.010 15878153

[B4] World Health Organization Global plan for artemisinin resistance containment (GPARC) 2011 World Health Organization, Geneva

[B5] World Health Organization Progress on the Containment of Artemisinin Tolerant Malaria Parasites in South-East Asia (ARCE) Initiative 2010 World Health Organization, Geneva

[B6] World Health Organization Strategic Plan to Strengthen Malaria Control and Elimination in the Greater Mekong Subregion: 2010–2014 2009 A Mekong Malaria Programme Partnership Initiative - Working Document, World Health Organization

[B7] World Health Organization Update on artemisinin resistance 2012

[B8] World Health Organization Malaria in the Greater Mekong Subregion: Regional and Country Profiles 2010 World Health Organization, Geneva

[B9] World Health Organization Regional action plan for malaria control and elimination in the western pacific (2010–2015) 2009 World Health Organization, Geneva

[B10] Incardona S Vong S Chiv L Lim P Nhem S Sem R Khim N Doung S Mercereau-Puijalon O Fandeur T Large-scale malaria survey in Cambodia: novel insights on species distribution and risk factors Malar J 2007 6 37 10.1186/1475-2875-6-37 17389041 PMC1847522

[B11] Tipmontree R Fungladda W Kaewkungwal J Tempongko MA Schelp FP Migrants and malaria risk factors: a study of the Thai-Myanmar border Southeast Asian J Trop Med Public Health 2009 40 1148 1157 20578448

[B12] Wongsrichanalai C Sirichaisinthop J Karwacki JJ Congpuong K Miller RS Pang L Thimasarn K Drug resistant malaria on the Thai-Myanmar and Thai-Cambodian borders Southeast Asian J Trop Med Public Health 2001 32 41 49 11485094

[B13] Meankaew P Kaewkungwal J Khamsiriwatchara A Khunthong P Singhasivanon P Satimai W Application of mobile-technology for disease and treatment monitoring of malaria in the "Better Border Healthcare Programme" Malar J 2010 9 237 10.1186/1475-2875-9-237 20723223 PMC2936405

[B14] Malaria Consortium and World Health Organization Proceedings from the workshop on monitoring and evaluation (M&E) indicators for the Bill & Melinda Gates supported project 2009 A strategy for the containment of artemisinin resistant malaria parasites in Southeast Asia

[B15] Khamsiriwatchara A Wangroongsarb P Thwing J Eliades J Satimai W Delacollette C Kaewkungwal J Respondent-driven sampling on the Thailand-Cambodia border. I. Can malaria cases be contained in mobile migrant workers? Malar J 2011 10 120 10.1186/1475-2875-10-120 PMC3116496 21554744

[B16] Lynch C Roper C The transit phase of migration: circulation of malaria and its multidrug-resistant forms in Africa PLoS Med 2011 8 e1001040 10.1371/journal.pmed.1001040 21655316 PMC3104977

[B17] Dondorp AM Yeung S White L Nguon C Day NP Socheat D von Seidlein L Artemisinin resistance: current status and scenarios for containment Nat Rev Microbiol 2010 8 272 280 20208550 10.1038/nrmicro2331

[B18] Chilundo B Sundby J Aanestad M Analysing the quality of routine malaria data in Mozambique Malar J 2004 3 3 10.1186/1475-2875-3-3 14998435 PMC395838

[B19] O'Sullivan M Kenilorea G Yamaguchi Y Bobogare A Losi L Atkinson JA Vallely A Whittaker M Tanner M Wijesinghe R Malaria elimination in Isabel Province Solomon Islands: establishing a surveillance-response system to prevent introduction and reintroduction of malaria. Malar J 2011 10 235 10.1186/1475-2875-10-235 PMC3175476 21834995

[B20] Mushi RT Chilimo W Contribution of Information and Communication Technologies to Malaria Control in Tanzania International Journal of Information Communication Technologies and Human Development 2011 3 52 60

[B21] Booman M Sharp BL Martin CL Manjate B La Grange JJ Durrheim DN Enhancing malaria control using a computerised management system in southern Africa Malar J 2003 2 13 10.1186/1475-2875-2-13 12816547 PMC161823

[B22] Chanda E Mukonka VM Mthembu D Kamuliwo M Coetzer S Shinondo CJ Using a geographical-information-system-based decision support to enhance malaria vector control in Zambia J Trop Med 2012 2012 363520 22548086 10.1155/2012/363520 PMC3323906

[B23] Booman M Durrheim DN La Grange K Martin C Mabuza AM Zitha A Mbokazi FM Fraser C Sharp BL Using a geographical information system to plan a malaria control programme in South Africa Bull World Health Organ 2000 78 1438 1444 11196490 PMC2560669

[B24] Shirayama Y Phompida S Shibuya K Geographic information system (GIS) maps and malaria control monitoring: intervention coverage and health outcome in distal villages of Khammouane province Laos. Malar J 2009 8 217 10.1186/1475-2875-8-217 PMC2754997 19772628

[B25] Country Coordinating Mechanism T Proposal Form, Round 10-Single Country Applicant, Section 3–5 2010 Malaria

